# Acute Respiratory Infection CD Module

**DOI:** 10.3201/eid0805.020024

**Published:** 2002-05

**Authors:** Patricia L. Riley, Elizabeth A. Downes, Malcolm P. Chikomo

**Affiliations:** * Centers for Disease Control and Prevention, Atlanta, GA, USA; † Emory University, Atlanta, GA, USA

The Acute Respiratory Infection (ARI) CD Module is the latest educational offering by The Wellcome Trust, a British-based charity. The module is part of the Topics in International Health CD-ROM series. The entire program is designed to provide accessible, current, high-quality information on tropical and international health. The material is directed towards students, teachers, health-care professionals, academics, and researchers in medicine and the life sciences. Each tutorial has been reviewed by at least two international subject experts.

The ARI module includes 11 separate tutorials ranging from etiology and risk factors to epidemiology and program management. An additional image collection of over 600 screens with accompanying text provides a useful bank of visual aids.

While the module covers all major ARIs, the focus is on conditions causing substantial illness and deaths in the developing world. Each tutorial averages 40 screens, with each individual screen featuring a variety of additional interactive tools, such as pop-up boxes and animated features. On average, tutorial post-tests follow every 6-10 screens. Although the content and information differ, each tutorial averages 2-4 hours to complete. Completing all 11 tutorials could take up to 50 hours.

Overall, the module succeeds in consolidating broad-reaching material and providing an authoritative presentation on ARI. Module strengths include the successful presentation of clinically current and well-researched information. (One possible exception is the management of ear problems. The module recommends irrigation of acute otitis media. This practice is highly questionable, and the accompanying illustrations minimize the major risks associated with this procedure in cases where the tympanic membrane has ruptured. The World Health Organization recommends “wicking.”) Each tutorial provides ample references, and the repetition of important themes helps reinforce important clinical and public health concepts. The graphic features and video inserts are equally useful. For example, the integration of animated graphics in the pathology tutorial to demonstrate the major steps of viral multiplication offers an innovative visual tool for mastering complex information.

On the other hand, the module suffers in its attempt to cover a large quantity of material in a similar manner. Not all the tutorials are of comparable complexity. While 40 screens may be adequate to discuss ARI prevention and control measures, other sections, such as pathology and respiratory defenses, would have benefited from more discussion. By trying to maintain uniform format of comparable length, parts of these tutorials are superficially summarized at the expense of a clear and in-depth discussion. Another weakness pertains to the varying levels of difficulty in the post-tests interspersed throughout the module. Some of the questions are challenging, whereas other post-tests could be completed correctly without ever having taken the tutorial. Minor typographical errors were noted throughout; however, they did not detract from the overall presentation.

More important issues pertain to the module’s intended audience. Although the material and clinical examples are designed to address ARIs in the developing world, they are not aimed at professionals from those countries. Rather, the module is better suited for health professionals from the industrialized world who have an interest in global health. The module would be far more practical had it been field-tested in those countries from which both clinical and public health examples were drawn.

The module requires additional software for accessing video clips, such as QuickTime or RealPlayer. The cost of the CD-ROM is also relatively high: $195.00 for institutions and $55 for students and individual purchasers. These two features limit the module’s practicality as a teaching device for health professionals in the developing world. Additionally, the module does not have the capability of multitasking with other software applications. The user must exit the module to use any other software, which limits the ability to access other references at the same time.

In summary, the Acute Respiratory Infection CD Module serves as a useful adjunctive teaching tool for both clinical and public health practitioners serving the developing world. It is not intended to replace the clinical component of provider training. While the material is comprehensive in scope, this aspect conversely leads to an uneven presentation in places. Given the required sophisticated software and cost of the CD, its usefulness for health professionals from the developing world is questionable.

